# The Impact of Symbolic and Non-Symbolic Quantity on Spatial Learning

**DOI:** 10.1371/journal.pone.0119395

**Published:** 2015-03-06

**Authors:** Koleen McCrink, Jennifer Galamba

**Affiliations:** Department of Psychology, Barnard College of Columbia University, New York, NY, United States of America; The University of Western Ontario, CANADA

## Abstract

An implicit mapping of number to space via a “mental number line” occurs automatically in adulthood. Here, we systematically explore the influence of differing representations of quantity (no quantity, non-symbolic magnitudes, and symbolic numbers) and directional flow of stimuli (random flow, left-to-right, or right-to-left) on learning and attention via a match-to-sample working memory task. When recalling a cognitively demanding string of spatial locations, subjects performed best when information was presented right-to-left. When non-symbolic or symbolic numerical arrays were embedded in these spatial locations, and mental number line congruency prompted, this effect was attenuated and in some cases reversed. In particular, low-performing female participants who viewed increasing non-symbolic number arrays paired with the spatial locations exhibited better recall for left-to-right directional flow information relative to right-to-left, and better processing for the left side of space relative to the right side of space. The presence of symbolic number during spatial learning enhanced recall to a greater degree than non-symbolic number—especially for female participants, and especially when cognitive load is high—and this difference was independent of directional flow of information. We conclude that quantity representations have the potential to scaffold spatial memory, but this potential is subtle, and mediated by the nature of the quantity and the gender and performance level of the learner.

## Introduction

Decades of experimental research have demonstrated that representations of number are linked to spatial locations. A widely supported example of this relationship is the Spatial Numerical Association of Response Codes (SNARC) effect, in which Western-educated individuals preferentially map smaller numbers to the left side of space, and larger to the right, in an ordered sequence [1]. This effect is attributed to a cognitive mapping of symbolic number to a spatial continuum, or internal *mental number line* [1, 2]. The mapping of number to space is found when individuals process symbolic numerals [1, 3], as well as sets of objects [4], which are represented by the non-symbolic Approximate Number System [5]. The bidirectional associations between spatial, numerical, and temporal cues can even be found in the earliest months of life [6, 7].

However, it is not simply number that gets mapped to space in the adult mind; SNARC-“like” effects have been documented for stimuli as variable as letters, luminance patches, months, and auditory pitch [8–10]. The common denominator of all these effects is that the dimension of interest is ordinal, either inherently (e.g., the increasing frequency of waves dictating the pitch of a sound) or via extensive training within the experiment [11, 12]. For example, even an arbitrary sequence of words—if trained to be placed into a specific order—leads adults to map the initial words to one side of space and final words to the other [11]. Thus it appears that placement along a horizontal spatial continuum is a spontaneous and natural manner for adults to structure information of many types.

The placement of information along a spatial continuum results in shifts of visuo-spatial attention. A peripheral detection paradigm has been used to assess the direction of participants’ attention when primed with Arabic numerals [13]. In the task, participants were asked to indicate the lateral location of a peripheral target after being primed with a relatively small (1 or 2) or relatively large (8 or 9) digit. Subjects responded more quickly to the left-side target when primed with the small number, and faster to the right when primed with a large number. These findings suggest that individuals shift their attention to the left or right, depending on the magnitude of the number with which they are primed.

Further evidence for shifts of attention comes from work using a line-bisection task, in which subjects are asked to indicate their perceived midpoint of a line flanked by Arabic numerals of differing magnitudes. Despite the fact that the attention of the subjects is never explicitly drawn to the flanking numbers, and the fact that the flanking numbers are irrelevant to the task demands, adults consistently demonstrate a spatial bias towards the larger number [14, 15]. This bias occurs independently of the lateral position of the larger flanker number (on the left side of the line or the right), is found for both non-symbolic and symbolic arrays, and is present as early as the preschool years [15]. The proposed explanation of this phenomenon is that because area (the spatial continuum) and number (the symbolic or non-symbolic flanker) are intertwined, the length proximate to the smaller flanking number is cognitively expanded, and occupies a larger amount of representational space than the area of the line that lies closest to the larger flanking number. The most concrete evidence for shifts of spatial attention comes from experiments that have utilized an eye tracker to document what happened when subjects were centrally presented with a large or small magnitude, followed by a target on the left or right side of the screen [4]. Subjects were faster to orient to left-side targets when primed with small sets of objects or small numerals, and right-side targets when primed with large sets or numerals.

The presence of a spatial aspect to numerical processing necessitates a consideration of how the gender of the subject may influence spatial-numerical interactions. Given that the parietal lobe is associated with the spatial representation of number [16] and that sex differences exist in amount of surface area in the parietal lobe [17], there is reason to expect that there may be sex differences in the spatial-numerical realm. Indeed, recent evidence suggests that not only do men exhibit more spatial-numerical linkage in the “gold standard” SNARC task (a parity task), but they also show this pattern in a battery of spatial-numeric tasks, such as number line estimation tasks and magnitude decision tasks [18].

The propensity to order information in a particular *directional* manner (e.g., left-to-right, or right-to-left) is influenced by the surrounding culture of the subject, primarily through the reading and writing directionality of the most-prominent language. For instance, the left-to-right spatial continuum documented in English-speaking adults is reversed or attenuated in right-to-left language cultures such as Hebrew, Farsi, or Arabic [1, 19, 20]. Iranians living in France, who learned to write from right-to-left, show a Westernized left-to-right mapping of space and number that is dependent upon the number of years since they left Iran [1], and illiterate Arabic-speakers do not exhibit a reliable SNARC effect [17]. In addition, young children count in a directional manner that is consistent with their culture’s linguistic flow [21], and preferentially learn about stimuli that are numerically and spatially related if that relation is consistent with the child’s experience in a culture [22, 23]. When monolingual English-speaking preschoolers were provided with numerical labels for spatial locations, they were better able to use that information if the mapping took place in a culturally consistent left-to-right fashion [22]. This effect extends to other ordered stimuli (such as letters of the alphabet), and preschoolers in Israel—whose language and cultural milieu are more right-to-left than left-to-right, though not exclusively—show the opposite effect (with better performance for labels mapped in a right-to-left manner) [23]. These findings with young pre-readers suggest that the directionality of SNARC and SNARC-like effects are inculcated even before the advent of self-directed and automatized reading, making it more likely that SNARC and SNARC-like effects are products of subtle and early environmental influences associated with an individual’s surrounding linguistic context.

The current investigation uses a classic visuo-spatial working memory task in which subjects must repeat back a series of previously-learned spatial locations until performance deteriorates [24, 25]. Previous studies have examined whether imposing structure on incoming spatial information facilitated recall in this type of task, by manipulating whether the sequence followed a rule in which each location occurred in the same column, row, or diagonal as the previous location [26]. Subjects were better able to recall these structured sequences compared to randomized sequences, indicating that they used spatial context to support learning and memory. We build upon these findings, and the body of work on culturally mediated spatial-numerical links, to investigate three central questions. First, how do differing types of numerical and non-numerical information support spatial memory? Second, does one’s cultural environment influence the propensity to learn from particular types of spatial structure? Finally, how do different types of numerical information *interact* with spatial flow to influence spatial memory?

In each of four experiments, subjects must view and then replicate an iterative series of sequentially highlighted spatial locations on a large grid using a computerized touchscreen. In each experiment there are three spatial flow types: left-to-right (each subsequent panel is highlighted in the same column or to the right of the previous panel), right-to-left (each subsequent panel is highlighted in the same column or to the left of the previous panel), or random (no structure to the spatial sequence.) The exact nature of the stimuli to be encoded, and the difficulty of the recall task, differs by experiment. Experiments 1 through 3A present the subjects with a chance to start with a very simple 1-location string that grows to a challenging 10-location string. The exact nature of the stimuli varies; spatial locations only were illuminated in Experiment 1, non-symbolic numerical arrays were embedded into the locations for Experiment 2, and symbolic numerals were embedded into the locations for Experiment 3A. Experiment 3B presented subjects with a more-difficult version of Experiment 3A, such that the subjects start the learning process with a longer initial string of spatial locations. These manipulations allowed us to examine whether adults exhibit better memory for spatial locations that are mapped to number in a fashion that is consistent with the MNL of these subjects (small numbers to the left side of space, and large numbers on the right), compared to a reversed manner (small numbers on the right side of space, large on the left) or inconsistent manner (with small number and large number equally likely on either side), and whether this process changes as a function of the type of stimuli to be processed (non-numerical, non-symbolic quantity, or symbolic quantity).

First, we predict a straightforward cognitive benefit of structure when encoding spatial information; structured spatial information will be more accurately recalled than randomly presented information. Second, we predict that left-to-right structure will be the most beneficial for learning, since it is the nature of the information most commonly consumed by these subjects. Third, we predict that the benefit of left-to-right spatial structure will be most pronounced when the spatial information is paired with increasing numerical information, because the left-to-right nature of the spatial flow is culturally congruent with the nature of the presented magnitude.

## Experiment 1: Spatial Location Encoding and Recall

In this first experiment, we aim to map the basic ability of adults to learn and recall from memory an increasing string of item locations. This experiment provides a baseline in the current paradigm as to how spatial structure impacts learning and memory, irrespective of concurrent numerical processing. Further, it allows us to examine whether the predicted advantages of culturally consistent spatial flow are number-specific. If there is a global benefit of spatial structure that conforms with the particular subject’s cultural norms (for these English speakers, a tendency for information to be presented or consumed from left-to-right), one would predict best performance for spatial information presented left-to-right, middling performance right-to-left, and worst performance for information presented indiscriminately. Given the fact that SNARC-like effects have been found for dimensions that are ordinal—but not necessarily numerical [8–10]—this is a distinct possibility. If, on the other hand, the benefit of culturally specific spatial structure only comes about when quantitative processing is invoked, we would expect an equal benefit for any spatial structure (LR, or RL) over non-structure (IND).

### Method

#### Ethics Statement

The following experiments were conducted after obtaining Institutional Review Board approval from Barnard College. All participants gave written informed consent before testing began.

### Subjects

32 subjects (16 female), naïve to the purpose of the experiment, were recruited from flyers and the Introductory Psychology subject pool. All subjects were screened for right-handedness and to determine if they were fluent in a language that possessed right-to-left directionality (e.g., Hebrew, Farsi, Arabic). An additional 2 subjects participated but were excluded from the results because they did not meet the language criteria. Although we did not gather exact ages from the subjects, the population tested in this and all subsequent experiments was comprised of undergraduate students from Columbia University, where the majority of students fall into the category of young adult (18–29 years of age).

### Stimuli

Visual stimuli consisted of slides with a 4x4 matrix of spatial locations created on Keynote Presentation Software. Superlab 4.5 software was used to present the stimuli on a MacBook Pro 15-inch laptop, with a MagicTouch touchscreen Model KTMT-1700W-USB-M added to record behavioral responses. At a viewing distance of approximately 40 cm, participants watched videos in which spatial locations were briefly illuminated (e.g., turned to gray from black) for 300 ms. The locations remained lit while new locations illuminated. Each spatial location was a box 12.4 cm wide, and 7.4 cm high, on a black background.

### Design

Each participant was given three versions of each of the three spatial flow types: left-to-right (LR, in which the series starts on the left side of the screen and ends on the right side), right-to-left (RL, in which the series starts on the right and ends on the left), and indiscriminate (IND, in which the series of spatial locations light up randomly around the screen.) The LR and RL spatial types were mirrors of each other, to make them directly comparable and control for any location-specific effects. This design yields 9 trials, each comprised of 55 subtrials, the number of trials it takes to get from the repetition of 1 panel (a short string) building up to 10 panels (a long string). Testing sessions for adult participants lasted approximately 25 minutes. The order of trials was counterbalanced to ensure each spatial flow subtype was presented first to approximately one-third of the participants.

### Procedure

Subjects were seated in front of a laptop, and were told they would be playing a spatial memory game. On-screen they saw the following instructions: “Learn the order of the lit up squares presented. When prompted, touch the center of the screen, and proceed to enter the previous order of the squares memory. Touch the screen to move on.” They then touched the screen, at which point the word “Learn!” was presented, followed by the first panel illumination. The word “Repeat!” was then presented, followed by a fixation cross in the center of the screen to ensure a neutral starting position before the subject indicated their answer, and then a blank black screen after the subject pushed the cross location. The computer subsequently recorded where and when the subject touched, timing out on the trial if the subject did not respond within 5 seconds.

### Results: Experiment 1

Subjects were given a score that aggregated their percentage correct in indicating the positions of the panels they saw during the learning sessions, pooled over each of the 3 trials per each spatial type (LR, RL, IND), and separated by String Length (T1—in which only 1 panel was presented for learning and repetition- through T10, in which 10 panels were presented). [S1 Dataset provides the dataset for this and all subsequent experiments.] For example, a subject who got 4/5, 5/5, and 3/5 panels correct for each of the LR 5-string trials—recall that each spatial type had three different configurations—would have a T5 score for LR of. 80. An ANOVA was conducted on the percentage correct response rate, with within-subjects factors of Spatial Type (LR, RL, IND), and String Length (1 through 10), and a between-subjects factor of gender (male, female). There was a significant overall main effect of spatial type (*F*(2,60) = 17.73, *p* <. 001, partial *η2* = .37); LR (90.8%, *SEM* = 1.6) and RL (92%, *SEM* = 1.6) were better than IND (86%, *SEM* = 2.0). There was also a significant main effect of String Length (*F*(9,270) = 35.69, *p* <. 001, partial *η2* = .54), as subjects’ performance decreased from the shorter length spans (98% at T1) to the longest ones (76% at T10). String Length interacted with Spatial Type (*F*(18,540) = 5.6, *p* <. 001, partial *η2* = .16); as would be expected, performance for all spatial types started off quite high (99%, 100%, and 96% T1 for LR, RL, and IND respectively), but tapered to different performance as the strings reached maximum length (79%, 85%, and 63% at T10 respectively; planned comparisons corrected for multiple comparisons reveal a difference between IND and the structured trials starting at 8 items). We calculated the slope of each subject’s best fitting line for their performance for each spatial type as the number of to-be-remembered items lengthened. A repeated-measures ANOVA with spatial type as the within-subjects factor, and gender as a between-subjects factor, reveals that the IND sub-condition slope is significantly steeper than LR or RL slopes (all *p*s <. 01, corrected for multiple comparisons).

Because one of our central questions is the role of culturally specific structure, we now turn to a more-specific general linear model. The LR and RL trials were designed to be mirrors of each other; thus, there cannot be an asymmetric influence of particular spatial factors specific to only one sub-condition (e.g., corners, or spatially-close transitions). An ANOVA over percentage correct with Spatial Type (LR, RL) and String Length (1 through 10) as within-subjects factors and gender (male, female) as a between-subjects factor found no main effect of spatial type or gender, and the expected main effect of String Length as subjects performed worse as the memory string lengthened (*F*(9,270) = 20.78, *p* <. 001, partial *η2* = .42). Slope analyses also reveal no significant effect of Spatial Type on learning through time.

**Fig 1:**
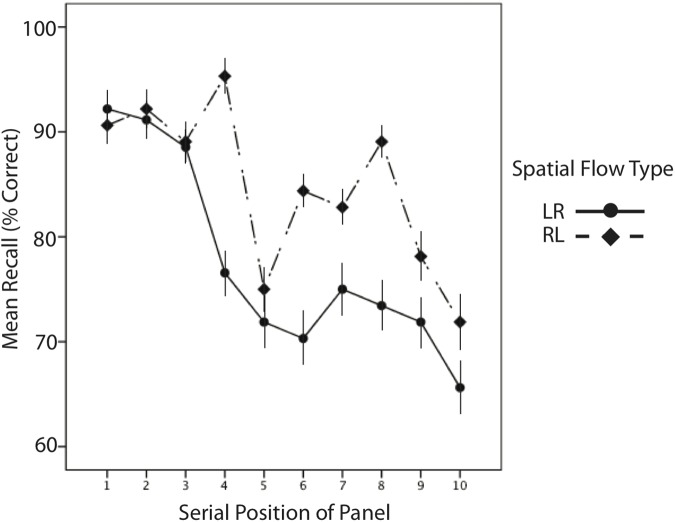
Effect of Spatial Flow on Spatial Location Recall for Experiment 1. Participants’ performance at each position in the 10-panel recall string (T10) for Experiment 1: Space Only, for the left-to-right structured and right-to-left structured spatial flow types. Error bars are the within-subject standard error of the mean (SEM).

One well-known finding in the literature is that when the cognitive system is under load the use of strategies and heuristics increases [27, 28]. For example, students faced with easy and hard arithmetic problems are more likely to employ a supporting strategy (such as finger counting) for hard problems, and this increases their accuracy relative to students who do not use strategies [29, 30]. One approximation of this load in our paradigm is T10, in which 10 locations need to be recalled and repeated on the touchscreen. For this experiment (and all subsequent experiments as well), we calculated serial position curves for T10, charting performance on the subjects’ repetition of the first panel, second panel, and so forth. (Note: Due to a programming error, T10 was disrupted mid-string for one of the three types of LR spatial sub-conditions. Accordingly, this particular type of LR trial—and its mirrored RL—trial are not factored into any analysis specifically on T10.) The subjects each receive a score for Position 1 through 10 in each Spatial Type sub-condition. An ANOVA over these scores with Spatial Type (LR, RL) and Serial Position (1^st^ through 10^th^) as within-subjects factors and gender (male, female) as between-subjects factors was conducted. There was an overall main effect of Spatial Type (*F*(1,30) = 5.9, *p* = .02, partial *η2* = .17), with subjects performing better overall in the RL trials (85%, *SEM* = 2.9) than LR (78%, *SEM* = 3.3; See Fig. 1). There is also a main effect of Serial Position (*F*(9,270) = 6.85, *p* <. 001, partial *η2* = .19); subjects exhibited better performance in the beginning part of the string that had been shown repeatedly, and worse performance when recalling the newer and less-frequently encoded spatial locations. A marginal interaction of Serial Position with Spatial Type (*p* = .09) indicates that this difference between RL and LR increases later in serial position. There were no main effects or interactions involving gender.

Another way to measure cognitive load is on an individual level, by calculating subjects’ overall performance and grouping them as High or Low performing based on a median split. To this end, we performed an ANOVA over subjects’ percentage correct, with Spatial Type (LR, RL, IND) as the within-subject factor and gender (male, female) and Performance Level (High, Low) as between-subjects factors, and followed up on significant interactions by using pairwise comparisons corrected for multiple comparisons. There was a significant main effect of Spatial Type (*F*(2,56) = 18.95, *p* <. 001, partial *η2* = .40), with LR (91%) and RL (92%) being similar to each other and higher than IND (86%). There was an interaction between Spatial Type and Performance Level (*F*(2,96) = 3.11, *p* = .05, partial *η2* = .10); for the Low performers, RL (87%) was significantly higher than LR (84%), which was higher than IND (78%), but the High performers exhibited equivalent performance for LR (97%), RL (97%), and IND (93%).

The fact that subjects were better at recalling the newer information in the string when it was presented in a right-to-left fashion opens the possibility that better overall processing for left-side information when recall becomes difficult may be driving this effect (a Left Visual Advantage, or LVA [31, 32]). As subjects moved from right to left, they were on the left side of the screen as they (recalled and) indicated their responses for the end of the spatial string. To test this, we computed performance as a function of spatial location, and gave participants an average score for the left-most and right-most columns for each spatial type. This was done for the whole experiment as well as on T10, since that is where recall is most difficult. A repeated-measures ANOVA with spatial type (IND, LR, RL) and side of screen (left, right) as within-subjects variables and gender as a between-subjects variable was conducted overall and for T10. If absolute position—and not simply the way information was flowing as it was learned—was influencing performance, we would expect to see a main effect of side. This was not the case for performance across the whole experiment (*F*(1,30) = 2.78, *p* = .11) or even selectively for T10 (*F*(1,30) = .01, *p* = .99), though there was the expected interaction between side and spatial type, as the LR and RL trials have their easiest-to-recall shorter and earlier strings on opposite sides of the screen.

### Discussion: Experiment 1

We can draw several conclusions from this experiment. The first is that this paradigm readily invokes adults’ propensity to draw upon spatial structure to support memory and recall of information. Performance was higher when subjects were able to narrow the range of possibilities of where to expect and recall new information. However, we did not find an overall positive impact of culturally specific information flow on memory and recall. In aggregate, subjects performed similarly when recalling information presented from left-to-right or right-to-left. Only when the cognitive system was under maximum load (a 10-item recall), or subjects were performing poorly, did the subtle but potentially informative impact of directional spatial structures come to light. The pattern of results indicates that it is actually a *right-to-left* flow which enhances recall of spatial locations. Thus we see a benefit for moving attention leftward while reconstructing challenging, previously learned information. This finding is not driven by asymmetric absolute encoding of particular spatial locations, or a general advantage for processing of the left field.

This space-only experiment can serve as a useful baseline for examining the impact of numerical information on spatial processing. In Experiment 2, we provide participants with non-symbolic arrays inside the to-be-recalled panels, and emphasize numerical magnitude by keeping the variables of area and perimeter of the entire array constant. The number of objects presented increases as the trials progress, moving from one item in the first panel to ten items in the tenth and final panel. This manipulation creates a spatial-numerical congruency in the LR spatial type, a spatial-numerical conflict in the RL spatial type, and leaves the IND spatial type indeterminate (e.g., congruent half the time, and incongruent the other half.) These differing levels of conflict lead to the hypothesis that, insofar as the subjects in our population possess a Western-oriented mental number line, LR spatial types will be processed and recalled better than RL spatial types. It is unclear what to expect of RL performance relative to IND; if conflict between space and number is too disruptive to recall, we may see similar performance. If the benefit of spatial structure trumps spatial-numerical conflict, we will observe better performance for RL relative to IND.

## Experiment 2: Non-Symbolic Number Encoded in Space

**Fig 2:**
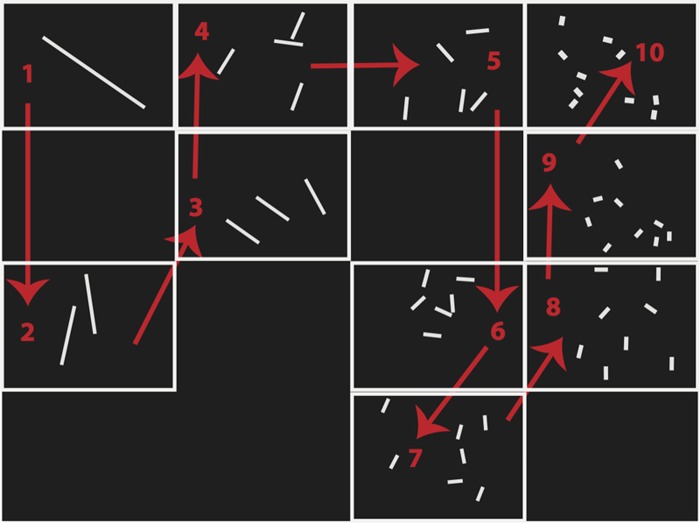
Experimental Schematic and Embedded Numerical Stimuli. Schematic of stimuli and spatial flow for Experiments 2, 3A, and 3B (left-to-right flow shown). In Experiment 2 the non-symbolic arrays were embedded in spatial locations as they appeared on the screen, and in Experiment 3A and 3B the symbolic numerals were embedded in the center of each location as it appeared.

The current experiment was identical to Experiment 1, with the exception of the stimuli encoded in the illuminated panels. In order to emphasize numerical magnitude, and de-emphasize correlated cues to quantity such as area and perimeter, each array had the same average area (1.4 cm^2^) and contour length (15.5 cm), with a 10% variability parameter, for the sets of 1 through 10 objects (see Fig. 2).

### Subjects

32 undergraduate students (16 female) from Columbia University, all right-handed and naïve to the purpose of the experiment were recruited via flyers and the Introductory Psychology subject pool. Subjects who were fluent in languages commonly written or read from right-to-left (e.g., Hebrew, Farsi, or Arabic) were excluded from the sample and replaced (2). An additional subject was run but excluded due to noise in the testing room.

### Results: Experiment 2

An ANOVA was conducted on the percentage correct response rate, with within-subjects factors of Spatial Type (LR, RL, IND), and String Length (1 through 10), and a between-subjects factor of gender (male, female). There was a significant main effect of spatial type (*F*(2,60) = 16.93, *p* <.001, partial *η2* = .36); LR (91.5%, *SEM* = 1.7) and RL (89.1%, *SEM* = 2.3) were better than IND (83.5%, *SEM* = 2.4). There was also a significant main effect of Trial (*F*(9,270) = 34.41, *p* <. 001, partial *η2* = .53), as subjects’ performance decreased from the shorter length spans (98% at T1) to the longest ones (73% at T10). Trial interacted with Spatial Type (*F*(18,540) = 8.01, *p* <. 001, partial *η2* = .21); as would be expected, performance for all spatial types started off quite high (98% at T1 for all LR, RL, and IND), but tapered to different performance as the strings reached maximum length (82%, 77%, and 59% at T10). Pairwise comparisons corrected for multiple comparisons indicate that starting at Trial 6, LR is significantly better than IND, and by Trial 9 both types of structured trials are better than IND. Trial also interacted with gender (*F*(9,270) = 3.3, *p* <. 01, partial *η2* = .10); by the time 5 items were to be remembered, female participants’ performance was significantly or marginally worse than male participants’. Female participants and male participants both started almost at ceiling on T1 (97% and 99% respectively), but decreased to 64% (female participants) and 82% (male participants). In addition to performance differing as the experiment progressed, there was also a main effect of gender (*F*(1,30) = 4.65, *p* = .039, partial *η2* = .13), with female participants (83.8%, *SEM* = 2.8) performing worse overall than male participants (92.3%, *SEM* = 2.8). There was also a marginal interaction of gender with Trial and Spatial Type (*F*(18,540) = 1.59, *p* = .05, partial *η2* = .05). This interaction reflects that when under the highest level of memory load female participants performed worse than men- but that this detriment was specific to the type of spatial flow (for LR trials, females were significantly worse at T5, T7, and T10; for RL—T3, T5, T5, T6, T7, T8, T9; for IND—T8 and T10.) This interaction is further explored below in the T10 analyses. We calculated the slope of each subject’s best fitting line of their performance for each spatial type at each string length. A repeated measures ANOVA with spatial type as the within-subjects factor, and gender as a between-subjects factor, reveals that the IND sub-condition slope is significantly steeper than LR or RL slopes (all *p*s <. 01, corrected for multiple comparisons). There was also a main effect of gender (*F*(1,30) = 4.85, *p* = .035, partial *η2* = .14); the slope of line of best fit for female participants’ performance is significantly steeper as the experiment progresses relative to male participants’.

**Fig 3:**
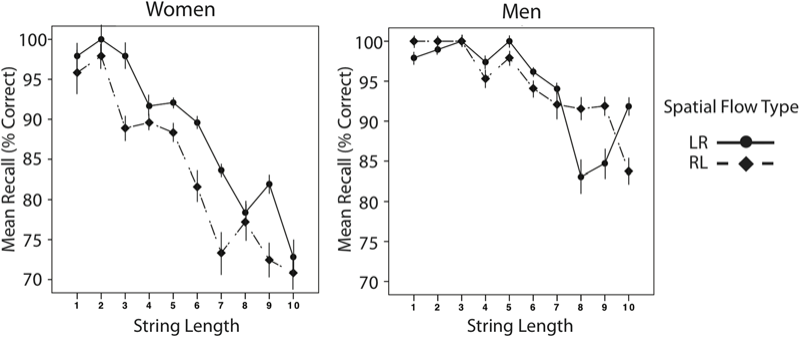
Spatial recall for Experiment 2: Non-symbolic Number Arrays. Female and male participants’ performance in Experiment 2: Non-Symbolic Number at each string length (1 panel up to 10 panels), for the left-to-right structured and right-to-left structured spatial flow types. Error bars are the within-subject SEM.

We now turn to the analyses including only the structured trials with spatial flow, and perform an ANOVA over percentage correct with Spatial Type (LR, RL) and String Length (1 through 10) as within-subjects factors and gender (male, female) as a between-subjects factor. There was a marginal overall effect of spatial type (*F*(1,30) = 3.66, *p* = .065, partial *η2* = .11), with LR performance overall at 91.5% vs. 89.1% for RL. This main effect is being driven by female participants’ performance, as reflected in a spatial type x gender interaction (*F*(1,30) = 4.48, *p* = .04, partial *η2* = .13). Female participants are performing better on LR (88.6%, *SEM* = 2.4) relative to RL (83.6%, *SEM* = 3.2) sub-conditions, but male participants perform similarly on both (94.4% and 94.7%) (See Fig. 3). There are also significant main effects of gender (*F*(1,30) = 4.92, *p* = .03, partial *η2* = .14) and String Length (*F*(9,270) = 19.43, *p* <. 001, partial *η2* = .39). Male participants outperformed female participants overall (94.5% (*SEM* = 2.7) as opposed to 86.1% (*SEM* = 2.7)). The expected main effect of String Length reflects a decrease in subjects’ performance as the memory string lengthened, from 98% at T1 to 80% at T10. The slope of lines fit to performance over time for each structured spatial sub-condition were entered into an ANOVA with Spatial Type (LR, RL) as a within-subject factor and gender (male, female) as a between-subjects factor; there were no main effects or interactions.

To analyze performance under the most challenging load, the data from the 10-string trials were analyzed, with each subject receiving a score for Position 1 through 10 in each Spatial Type sub-condition. An ANOVA over these scores with Spatial Type (LR, RL) and Serial Position (1^st^ through 10^th^) as within-subjects factors and gender (male, female) as between-subjects factors was conducted. There is a main effect of Serial Position (*F*(9,270) = 12.92, *p* <. 001, partial *η2* = .30); subjects exhibited better performance in the beginning part of the string that had been repeatedly shown up to this point, and worse performance in the newer information they had less experience with. There was also a main effect of gender, with male participants (88%, *SEM* = 4.8) outperforming female participants (70.7%, *SEM* = 4.8) at T10. There was a significant interaction between Serial Position, gender, and Spatial Type (*F*(9,270) = 1.95, *p* = .046, partial *η2* = .06). Female participants exhibited higher performance for the beginning of the string in LR sub-conditions relative to RL sub-conditions, an effect not found in the male participants’ performance. Indeed, male participants experience this benefit of LR mainly at the tail end of the string.

It is possible that these differences in use of spatial information flow are being driven by performance, not gender (which is confounded with overall performance here.) To examine this, and look as we did in Experiment 1 at how high- and low-performing subjects used different types of spatial structure, we conducted an ANOVA with Spatial Type (LR, RL, IND) as a within-subject factor and gender (male, female) and Performance Level (High, Low) as between-subjects’ factors. There is a main effect of Spatial Type (*F*(2,56) = 19.97, *p* <. 001, partial *η2* = .42), with LR (92%) and RL (91%) similarly high relative to IND (84%). Spatial Type interacted only marginally with gender (*F*(2,56) = 2.49, *p* = .09, partial *η2* = .08), though planned contrasts find a significant quadratic trend (*p* = .03), indicating that the relationship between the RL trials and other spatial types differed as a function of gender. For females, RL (87%) did not differ from LR (91%) or IND (83%), which were significantly different from each other. But for males, RL 94%) and LR (93%) were similar and both differed from IND (85%). Spatial Type also interacted with Performance Level (*F*(2,56) = 6.78, *p* <. 01, partial *η2* = .20). High performers did not exhibit performance modulation as a function of Spatial Type (LR 98%, RL 97%, IND 94%). Low performers saw a significant and equal benefit for both LR (86%) and RL (85%) relative to IND (74%).

To examine whether this benefit was due to spatial position (and not necessarily the flow of information), we coded performance for the extreme left and right column positions for the overall experiment as well as within T10. A repeated-measures ANOVA with spatial type (IND, LR, RL), position (left-side, right-side) as within subject variables and gender (male, female) as a between-subjects variable was conducted for each of these measures. As with the previous two experiments, there was no main effect for side in either the overall measures or the T10-specific measure. However, side did interact significantly with gender in both the overall and T10 ANOVAs (*F*s(1,30) = 4.12, 5.42, *p*s = .05,. 03 respectively). Follow-up analyses indicate that female participants were slightly better at recalling the left side of the screen relative to the right, whereas male participants showed the opposite tendency. For the experiment overall, female participants performed at 80% when information was left-side and 76% right-side, and male participants performed at 88% right-side and 85% left-side. At T10, female participants performed at 68% left-side and 64% right-side, male participants 88% right-side and 79% left-side. Although none of these individual comparisons reach significance when doing post-hoc tests (corrected for multiple comparisons), the interactions in the overall analyses indicate that it is the *relative* distribution that is notable.

### Discussion: Experiment 2

In this experiment, we presented increasing quantity information—with non-symbolic number emphasized—in a series of spatial locations that needed to be encoded and replicated. The panels appeared in an indiscriminate, left-to-right, or right-to-left manner, creating patterns that were neutral, congruent, or conflicting with the conventional MNL. Several main findings emerged. First, as expected, subjects performed much better at recalling long strings of information when it was structured (spatial flow conditions > indiscriminate), minimal (short strings > long strings), and presented repeatedly (previously seen information > relatively new information). Second, male participants outperformed female participants overall—especially as cognitive load increased. Third, we observe here an emergence of a benefit for spatial-numerical congruency. There was an overall trend for left-to-right spatial flow to increase recall, driven mainly by poor-performing female participants, who performed better overall in left-to-right spatial type trials relative to unstructured trials, but did not show this same benefit for right-to-left trials. It is only at maximal load (e.g., ten spatial locations need to be recalled, and some of this information is relatively new and has not been repeated) that we observe male participants exhibiting a benefit for information presented with spatial-numerical congruency. Finally, we see some evidence for preferential encoding of absolute spatial location (irrespective of MNL congruency or conflict); female participants were slightly better at remembering left-side information whereas male participants were slightly better at remembering right-side information.

The gender differences here were subtle but pervasive. Female participants’ performance was higher in left-to-right spatial sub-conditions relative to right-to-left conditions for every memory string length, something not observed with the male participants in this study. This raises an interesting dichotomy in performance; the women appear to be more attentive to the numerical information and spatial flow than the men, yet the men outperform the women. One possibility for why this may be relates to stereotype threat [33]. Although we did not deliberately prime subjects with gender-related information, the female participants in Experiment 2 may implicitly categorize the study as one pertaining to mathematics (a domain in which female participants are stereotypically weaker) upon seeing the quantity-related information, and experience lower confidence and subsequent performance. This well-documented phenomenon in the literature is often used to explain gender discrepancies on mathematical tasks that arise in the real world [34, 35] and the laboratory [36, 37] (although this is not the only view[38]). A second and related possibility is that subjects reach for “scaffolds” based in spatial flow only when faced with information they find challenging. The difference between high and low performers in terms of their overall use of any spatial structure provides some support for this hypothesis, although it cannot explain the influence of gender on this interaction. If the nature of the stimuli in this particular experiment resulted in them being construed by the female participants who are attentive to their already-poor performance as more mathematical and therefore more difficult, this may lead to decreased performance, and increased use of spatial-numerical strategies to enhance their performance. Finally, it may be the case that female participants are simply more attuned to the MNL than men, and as a result experience more interference from a mismatch between the MNL and the spatial-numerical sequences presented.

In the next experiment, we embedded the spatial locations with information that is explicitly numerical—Arabic numerals—to address whether the *type* of numerical information matters in a spatial memory task. Nearly all studies showing a link between quantity and space have utilized symbols, and suggest that the directional flow benefit is thought to be influenced by the cultural milieu of the subject, since culture-specific reading direction is highly symbolic, involving letters and numbers. The use of numerals embedded in the spatial panels in this experiment allows us to test several outstanding hypotheses. First, spatial information that has symbolic numerals embedded in it should be the most likely to elicit spatial-numerical associations; again we propose that sub-conditions with congruent spatial-numerical flow (e.g., LR) should be easier to learn and recall than those that are in conflict (RL) or randomly associated with (IND) the MNL. Although we have seen some evidence thus far for directional-specific learning benefits in the previous experiments, it was either in the opposite direction than predicted (the RL benefit for space-only learning), or specific only to a subset of subjects (a LR benefit for low-performing female participants when viewing non-symbolic number embedded in space.) Second, the use of symbolic number makes the mathematical nature of the task even more explicit to the subjects.

## Experiment 3A: Symbolic Number Encoded in Space

The current experiment was identical to Experiments 1 and 2, with the exception of the stimuli encoded in the presented panels. Instead of blank panels or numerical arrays, subjects saw the Arabic numerals 1 through 10 presented in the spatial locations.

### Subjects

32 undergraduate students (16 female) from Columbia University, all right-handed and naïve to the purpose of the experiment, were recruited via flyers and the Introductory Psychology subject pool. Subjects who were fluent in languages commonly written or read from right-to-left (e.g., Hebrew, Farsi, or Arabic) were excluded from the sample and replaced (1).

### Results: Experiment 3A

As before, subjects were given a score that aggregated their percentage correct in indicating the positions of the panels they saw during the learning sessions, pooled over each of the sub-trials per each sub-condition (LR, RL, IND). An ANOVA was conducted on the percentage correct response rate, with within-subjects factors of Spatial Type (LR, RL, IND), and String Length (1 through 10), and a between-subjects factor of gender (male, female). There was a significant main effect of spatial type (*F*(2,60) = 13.66, *p* <. 001, partial *η2* = .31); LR (95.0%, *SEM* = .7) and RL (95.6%, *SEM* = .7) were better than IND (91.0%, *SEM* = 1.2). There was also a significant main effect of String Length (*F*(9,270) = 39.45, *p* <. 001, partial *η2* = .57), as subjects’ performance decreased from the shorter length spans (100% at T1) to the longest ones (85% at T10). String Length interacted with Spatial Type (*F*(18,540) = 3.56, *p* <. 001, partial *η2* = .11); as would be expected, performance for all spatial types started off quite high (100%, 100%, and 99% T1 for LR, RL, and IND respectively), but tapered to different performance as the strings reached maximum length (89%, 91%, and 76% at T10, with a significant benefit for structured trials in T9 and T10). We now turn to analyses over only the structured sub-conditions (LR, RL). An ANOVA over percentage correct with Spatial Type (LR, RL) and String Length (1 through 10) as a within-subjects factor and gender (male, female) as a between-subjects factor found no main effect of spatial type or gender, or interactions with these variables. There was only the expected main effect of String Length as subjects performed worse as the memory string lengthened (*F*(9,270) = 25.72, *p* <. 001, partial *η2* = .46).

An ANOVA over T10 scores, targeted for the spatial flow conditions of LR and RL, calculated at each serial position with Spatial Type (LR, RL) and Serial Position (1^st^ through 10^th^) as within-subjects factors and gender (male, female) as between-subjects factors, was conducted. There was no overall main effect of Spatial Type, with subjects performing similarly overall in the LR T10 (89.2%, *SEM* = 1.7) and RL (90.6%, *SEM* = 1.8). There is a main effect of Serial Position (*F*(9,270) = 9.5, *p* <. 001, partial *η2* = .24); subjects exhibited better performance in the over-learned beginning part of the string, and worse performance in the end section. Spatial Type interacted with Serial Position (*F*(9,270) = 2.62, *p* = .01, partial *η2* = .08), and did so as a cubic interaction (as indicated by post-hoc planned contrasts); subjects performed better in RL spatial types when recalling the spatial position of panels that appeared at the beginning and end of the string, but were better in LR sub-conditions for recalling panels that happened in the middle of the string.

An ANOVA over percentage correct with Spatial Type (LR, RL, IND) as a within-subject factor and gender (male, female) and Performance Level (High, Low) was conducted in order to assess the impact of overall prowess on the use of spatial structure. There was a significant main effect of Spatial Type (*F*(2,56) = 18.04, *p* <. 001, partial *η2* = .39), with LR (95%) and RL (96%) performance similar and higher than IND (91%). Spatial Type also interacted with Performance Level (*F*(2,56) = 9.65, *p* <.001, partial *η2* = .26); for low performers, but not high, spatial structure was beneficial (High Performance: LR 97%, RL 98%, IND 97%; Low Performance: LR 93%, RL 93%, IND 85%).

To examine whether there was any influence of absolute spatial position (and not necessarily flow of information), we coded performance for the extreme left and right column positions for the overall experiment as well as within T10. A repeated-measures ANOVA with spatial type (IND, LR, RL), and position (left-side, right-side) as within subject variables and gender (male, female) as a between-subjects variable was conducted for the experiment overall as well as T10 only. In both cases, there was no main effect of side, and no meaningful significant interactions between side and any variable.

### Discussion: Experiment 3A

In this experiment, the subjects were tasked with recalling increasingly difficult strings of spatial locations, containing ascending symbolic numbers that were either in accordance with their conventional MNL (left-to-right spatial flow), in conflict (right-to-left spatial flow), or neutral (indiscriminate consistent flow). We again observe the finding that spatial structure supports recall—especially for poor-performing subjects—and longer strings are harder to remember than shorter strings. However, there was no clear evidence of directional flow impacting learning or memory, or spatial-numerical congruency assisting in encoding of information for later recall. The only notable interaction in the data occurred at the longest and most-challenging trial, and it does not support a theory that posits better memory for MNL congruency or familiar spatial flow; left-to-right spatial flow supported better recall of information for the middle of the sequence, and right-to-left spatial flow supported better recall of the beginning and end of the sequence. Finally, unlike in the previous experiment, in which number was emphasized (non-symbolically), male and female participants performed similarly.

The similar performance between male participants and female participants on this experiment is informative, especially in light of the findings from the non-symbolic number manipulation. Performance for both genders here was statistically comparable, and high. If one compares data from the two experiments, performance here was statistically higher than the previous experiment (94% vs. 88%, *p* <. 01), and an interaction of experiment and gender confirms this was carried by the female participants, who saw a nearly 10% performance bump when symbolic number was used instead of non-symbolic arrays (compared to a 1% increase for men.)

The main conclusion from this experiment is that symbolic number in general is an especially powerful tool for scaffolding spatial memory, and this is independent of the interaction between symbolic number and spatial flow of the information, and absolute placement of space and number. This was noticeable, anecdotally, as the subjects were run in our laboratory; they did not hesitate to repeat the strings in the beginning of the experiment, were less likely to ask the experimenters to repeat the instructions, and mastered the task requirements more quickly than subjects in the other condition. It was confirmed quantitatively by a boost for overall performance compared to the previous experiment, especially for female participants.

The central question of interest going into this experiment was whether embedding symbols into spatial locations would impact spatial memory, and whether this differed as a function of MNL congruency. Subjects did extremely well overall, with 95% CIs encompassing perfect performance for the majority of the trial string lengths. In order to create a scenario in which symbolic numbers were still embedded, but overall performance decreases, we designed a final experiment in which each trial started with a 5-panel string. In this way, the beginning part of the sequence is not overlearned by the time subjects arrive at the longer string, and they must master relatively new information alongside incoming information. If the adults draw on MNL congruency only under a challenging memory load, and that is why we do not observe a similar impact of spatial flow in Experiment 3A, then the added difficulty of this new experiment may result in similar results to Experiment 2.

## Experiment 3B: Symbolic Number—High Memory Load

The current experiment was identical to Experiment 3A, with the exception of the initial string length at the beginning of each trial. Instead of starting with the viewing and recall of a single panel, the string length started at 5 panels, in which the symbolic numerals 1 through 5 were embedded.

### Subjects

32 undergraduate students (16 female) from Columbia University, screened for right-handedness, were recruited via flyers and the Introductory Psychology subject pool. A subject who was fluent in a language written or read from right-to-left (Hebrew) was excluded from the sample and replaced.

### Results: Experiment 3B

Subjects were given a score that aggregated their percentage correct in indicating the positions of the panels they saw during the learning sessions, pooled over each of the sub-trials per each sub-condition (LR, RL, IND). An ANOVA was conducted on the percentage correct response rate, with within-subjects factors of Spatial Type (LR, RL, IND), and String Length (5 through 10), and a between-subjects factor of gender (male, female). There was a significant main effect of spatial type (*F*(2,60) = 19.92, *p* <. 001, partial *η2* = .39); LR (86.4%, *SEM* = 1.9) and RL (88%, *SEM* = 1.9) were better than IND (75.9%, *SEM* = 2.6). There was also a significant main effect of String Length (*F*(5,150) = 13.43, *p* <. 001, partial *η2* = .31), as subjects’ performance decreased from the shorter length spans (87% at T5) to the longest ones (77% at T10). There was also a significant main effect of gender (*F*(1,30) = 11.94, p = .002, partial *η2* = .29); male participants (89.8%) performed significantly better than female participants (77.1%). We now turn to analyses over only the structured sub-conditions (LR, RL). An ANOVA over percentage correct with Spatial Type (LR, RL) as a within-subjects factor and gender (male, female) as a between-subjects factor found no main effect of spatial type, or interactions with this variable. There was a significant main effect of gender (*F*(1,30) = 11.11, *p* = .002, partial *η2* = .27). There was also the expected main effect of String Length as subjects performed worse as the memory string lengthened (*F*(5,150) = 10.69, *p* <. 001, partial *η2* = .26).

For this experiment, we are particularly interested in both T5—which reflects the processing of a relatively-large set of new information in which flow cannot be anticipated—as well as T10, which reflects a high cognitive load, but over information which is practiced and whose flow is able to be anticipated. Two ANOVAs over T5 and T10 scores, targeted for the spatial flow conditions of LR and RL, calculated at each serial position with Spatial Type (LR, RL) and Serial Position (1^st^ through 5^th^, or 1^st^ through 10^th^) as within-subjects factors and gender (male, female) as between-subjects factors, were conducted. In the ANOVA over T5 scores, there was no overall main effect of Spatial Type, with subjects performing similarly overall in the LR T5 (91.5%, *SEM* = 2) and RL T5 (88.3%, *SEM* = 2.1). There is a main effect of Serial Position (*F*(4,120) = 10.94, *p* <. 001, partial *η2* = .27), and an interaction between Serial Position and Gender (*F*(4,120) = 5.11, *p* = .001, partial *η2* = .15). Subjects exhibited similarly high performance for the first 3 panels (95%, 95%, and 91%), and worse performance for the last 2 (84% for both). Female participants exhibited a relatively steep decline in performance from the first panel recall to the final panel recall in T5 (94% to 74%), whereas men’s performance did not decline with serial position (97% to 95%). This difference is also captured by an overall main effect of gender (*F*(1,30) = 16.61, *p* <. 001, partial *η2* = .36), with male participants scoring significantly higher than female participants (96% vs. 84%). Performance patterns in T10 were similar, with male participants outperforming female participants, earlier parts of the string recalled better than later parts, and female participants’ performance declining for the later serial positions whereas men’s performance remained steady. The only notable difference was a marginal interaction of serial position with spatial type (*p* = .07); performance in LR started off higher than RL (95% vs. 90%), but ultimately ended lower (73% vs. 79%).

An ANOVA over percentage correct with Spatial Type (LR, RL, IND) as a within-subject factor and gender (male, female) and Performance Level (High, Low) was conducted in order to assess the impact of overall prowess on the use of spatial structure. There was a significant main effect of Spatial Type (*F*(2,56) = 16.57, *p* <. 001, partial *η2* = .37), with LR (87%) and RL (90%) performance similar and higher than IND (77%). Spatial Type also interacted with Performance Level (*F*(2,56) = 3.27, *p* = .045, partial *η2* = .11); for low performers, but not high, spatial structure was beneficial (High Performance: LR 94%, RL 95%, IND 88%; Low Performance: LR 80%, RL 84%, IND 65%).

To examine whether there was any influence of absolute spatial position (and not necessarily flow of information), we coded performance for the extreme left and right column positions for the overall experiment as well as within T10. A repeated-measures ANOVA with spatial type (IND, LR, RL), and position (left-side, right-side) as within subject variables and gender (male, female) as a between-subjects variable was conducted for the experiment overall as well as T10 only. In both cases, there was no main effect of side, and no meaningful significant interactions with the side variable.

### Discussion: Experiment 3B

In this experiment, the difficulty of the experiment was enhanced by lengthening the initial number of panel locations to be recalled by the subjects, from a single location to 5. This manipulation was successful in bringing down performance to levels comparable to Experiments 1 and 2, to examine the effects of spatial-numerical congruency on memory under conditions of similar difficulty. We observed no overall effects of spatial directional flow, or the resultant MNL congruency/incongruency, on performance. When subjects were asked to recall a very long string of locations, they showed a trend to better recall the initial information if the spatial flow was from left-to-right, and final information if spatial flow was right-to-left. Similar to the previous experiments, there was no difference in memory for spatial locations based on which side of the screen they appeared on. Subjects who were relatively poor-performing were more likely to benefit from spatial structure than high-performing subjects. We again found gender differences; female participants performed worse than male participants, and did so under conditions of heavy cognitive load (e.g., when tasked with recalling newer information, or longer spatial strings).

## Overall Analyses

To pull out overall patterns that emerge from this set of experiments taken as a whole, and to statistically quantify any differences between experiments, we now turn to analyses in which the experiments are combined in a single model.

### Influence of Structured vs. Unstructured Information on Performance

**Fig 4:**
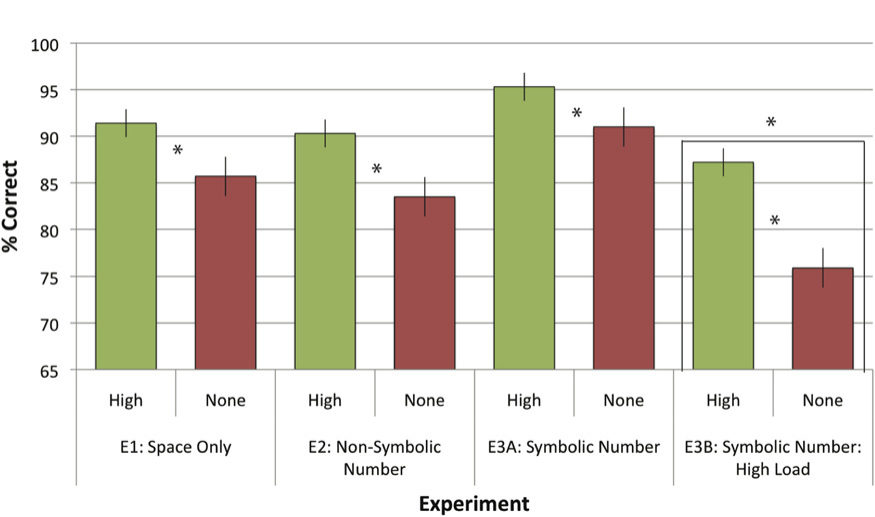
Recall performance in Structured vs. Non-Structured Sequences. Mean recall performance (% correct) in each experiment, contrasting a composite score for structured (LR and RL) trials in green vs. random structure (IND) trials in red. Error bars reflect the between-subjects SEM. The benefit of structure was apparent in all experiments (asterisks indicate a significant difference with an alpha level of. 05), and significantly greater in the cognitively demanding symbolic number condition.

In order to quantify the impact of spatial structure per se in our paradigm, and how it interacts with the nature of the stimuli, the subjects were given an aggregate score of their performance for spatial type sub-conditions which exhibited spatial structure (LR and RL). These scores, along with those for the non-structured IND spatial type, were entered into a repeated-measures ANOVA with Structure Type (Structured, Non-Structured) as a within-subjects factor and Gender (male, female) and Experiment (1, 2, 3A, 3B) as a between-subjects factor. There was a main effect of structure (*F*(1,120) = 91.53, *p* <. 001, partial *η2* = .43), with a successful recall rate of 91% in structured trials (*SEM* = .8) overall and 84% in unstructured trials (*SEM* = 1.1). There was a significant effect of gender (*F*(1,120) = 11.11, *p* = .001, partial *η2* = .09), with female participants (85%, *SEM* = 1.2) performing worse overall than male participants (90%, *SEM* = 1.2; see Fig. 3). There was also a main effect of experiment (*F*(3,120) = 7.62, *p* <. 001, partial *η2* = .16); planned comparisons corrected for multiple comparisons indicate that performance for the Symbolic Number experiment (93.2%) was slightly higher than that in Non-Symbolic Number (86.9%, *p* = .06) and the Symbolic Number High Load (81.8%, *p* <. 01) with middling performance in the Space-Only experiment (88.6%, all SEMs = 1.7, p = .33). Structure interacted with Experiment significantly (*F*(3,120) = 4.9, *p* = .003, partial *η2* = .11); the benefit of structure was greater for the Symbolic Number High Load (a 12% benefit) compared to Space Only (6%, *p* = .09), Symbolic Number (4%, *p* = .002), and Non-Symbolic Number (7%, *p* = .02; see Fig. 4.) Gender interacted with Experiment (*F*(3,120) = 3.25, *p* = .02, partial *η2* = .08), because male participants and female participants had different overall performance depending on the stimuli embedded in the panels. Female participants were significantly better when panels had Symbolic Number overall (91%), compared to Non-Symbolic Number and Symbolic Number High Load (78% each), and middling at Space Only (85%). Men’s performance remained statistically identical (87%, 89%, 91%, 90% across the 4 experiments).

### Influence of Absolute Spatial Position on Performance

Subjects were scored for their performance in recalling the left-most and right-most side of the screen during each trial. This is of interest because one possible influence on the current results is that subjects may exhibit a Left Visual Advantage (LVA: [26]) and excel at processing the left side of space, conflating a spatial flow benefit at the end of a LR or RL string with a generic screen-side benefit. Further, this effect may be exaggerated when symbolic stimuli—which can orient attention to the left or right side of space—are processed [13, 15]. A repeated-measures ANOVA over these scores with Side (Left, Right) and Spatial Type (LR, RL, IND) as within-subjects variables and gender (male, female) and Experiment (1, 2, 3A, 3B) as between-subjects variables was conducted. (Only those statistics pertaining to the variable of Side will be reported here, since the other analyses in this section capture other aspects of performance unrelated to Side.) There was no overall main effect of side; subjects similarly recalled left-side panels (85%) than right-side (84%). Gender did interact with visual side recall (*F*(1,90) = 3.72, *p* = .056, partial *η2* = .03); female participants were slightly better at recalling items on the left side of the screen (83% vs. 80% for the right side), whereas male participants were equally good at recalling items on both sides of the screen (88% for both sides). T10 of the IND spatial type—the most-cognitively demanding string to recall—was analyzed in a similar fashion (percentage correct when recalling the leftmost and rightmost panels during this long string). In this case, we find no significant effects of visual side. However, there was again an interaction between visual side and gender (*F*(1,120) = 3.99, *p* = .048, partial *η2* = .03), with female participants showing a benefit for left-side information over right-side information (77% vs. 75%, respectively) and male participants the opposite (82% vs. 85%). Thus we find partial support for LVA in this paradigm; both overall and under high cognitive load female participants, but not men, exhibit a (relative) LVA when processing the most-extreme sides of visual field.

**Fig 5:**
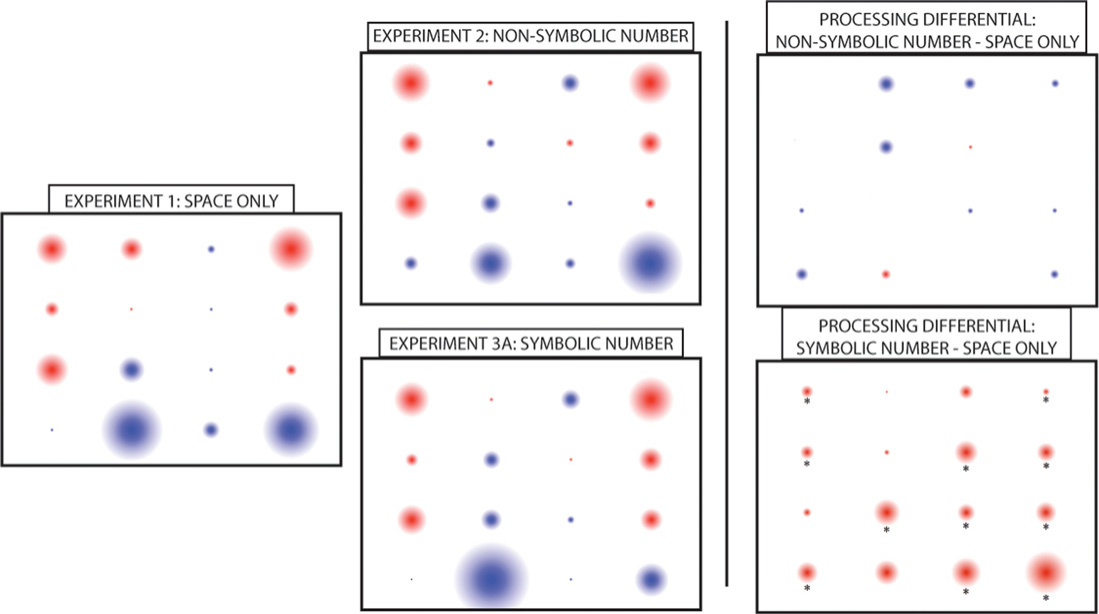
Overall Spatial Processing by Location and Quantity Type. Presented is a depiction of the relative strengths and weakness of spatial processing at each position on the screen. The diameter of the circle plots the deviation of that position to the group mean (e.g., its z-score), with positive scores in red reflecting better processing than average and negative scores in blue reflecting worse processing than average. The means for Experiment 1 were subtracted from those for Experiment 2 and 3A, the difference score normalized, and then plotted to provide the reader with a sense of where and under what numerical conditions spatial processing was enhanced or depressed. Processing—as measured by later recall—was best for upper corners and sides of the screen, worst for the bottom of the screen, and enhanced overall when symbolic numbers were concurrently presented in the spatial locations. Asterisks reflect a statistically significant recall difference between the two experiments, *p* <. 05, adjusted for multiple comparisons.

To provide a sense of spatial processing at each particular location in the 4x4 grid, we entered percentage correct at each location for Experiments 1–3A, collapsed over spatial flow type (Positions 1 through 16 on the grid, as one counts from left to right), into a repeated-measures ANOVA with Experiment and gender as between-subjects factors. (Because Experiment 3B had slightly different design characteristics, with some positions shown less frequently than in the other experiments, we could not make a direct comparison for this particular analysis.) We will outline here only effects pertaining to or meaningfully interacting with Spatial Position. There was a significant main effect of Spatial Position (*F*(15,1350) = 56.2, *p* <. 001, partial *η2* = .38), with some positions—mainly those on the lateral edges of the screen—eliciting higher recall than others. Spatial Position interacted with gender (*F*(15,1350) = 1.85, *p* = .03, partial *η2* = .02) as well as with gender and experiment simultaneously (*F*(30, 1350) = 1.92, *p* = .002, partial *η2* = .04). A follow-up repeated-measures ANOVA over spatial position performance for each experimental comparison (considering Space Only vs. Non-Symbolic Number, Space Only vs. Symbolic Number), with gender and experiment as between-subjects factors, reveals this is due to female participants in the non-symbolic experiment (Experiment 2) showing decreased performance at positions 2 and 6 relative to the baseline of Experiment 1. By contrast, performance was significantly better at the majority of spatial locations when symbolic stimuli were embedded in the spatial locations for Experiment 3A. In addition, there were no interactions with gender for this contrast of Symbolic Number vs. Space Only. Fig. 5 provides more information as to the relative strengths and weakness of spatial processing at each position for each experiment.

### Influence of Spatial Information Flow on Performance

**Fig 6:**
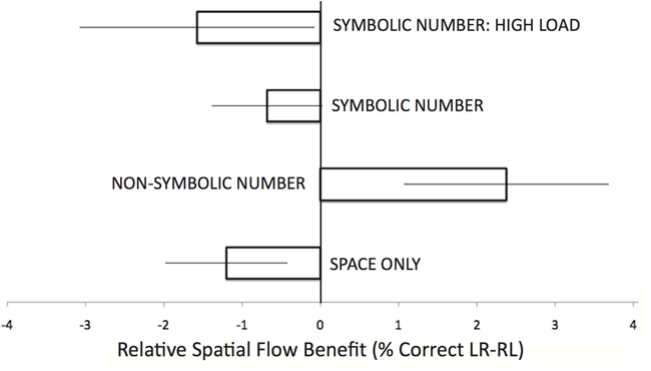
Impact of spatial flow type on recall. Participants’ performance differential for the two horizontal spatial flow types (LR—RL). The asterisk reflects a significant difference between the experiments at an alpha level of. 05, and error bars depicted are the SEM of each group.

An overarching hypothesis for this investigation was that the if the incoming flow of spatial information is familiar to the subject, such as a the left-to-right flow often experienced when reading or writing, the subject would experience a learning and recall benefit for that spatial type. The subjects scores for each spatial flow type for each string length was placed into a repeated-measures ANOVA with Spatial Type (LR, RL) and String Length (1 through 10) as a within-subject factor and gender (male, female) and experiment (1, 2, 3A) as between-subjects factors. (Experiment 3B was excluded due to design differences in string length.) There was no overall effect of Spatial Type; subjects performed identically overall in LR and RL (91%, *SEM*s = .8). Spatial Type interacted with Experiment (*F*(2,90) = 4.18, *p* = .02, partial *η2* = .09); although each experiment had no significant difference between performance at spatial flow types, the distribution was different as a function of the stimuli embedded in the panels (see Fig. 6), with slightly better LR performance in the Non-Symbolic Number experiment (91.5% vs. 89.1%), and slightly better RL performance in the other experiments (92.0% vs. 90.8% in Space Only, 95.6% vs. 95.0% in Symbolic Number). Post-hoc tests, corrected for multiple comparisons, over the differences scores in a one-way ANOVA reveal that the Non-Symbolic LR differential was significantly different from the Space Only (*p* = .03) and marginally different from the Symbolic Number (*p* = .085). There was also an interaction between Spatial Type, Experiment, and gender (*F*(2,90) = 2.9, *p* = .06, partial *η2* = .06), reflecting the gender differences found in spatial flow processing in the Non-Symbolic experiment.

To see how the pattern of learning unfolded over time, as the experiments grew from less- to more-challenging recall scenarios, we examine effects involving String Length. There was the expected main effect of String Length (*F*(9,810) = 60.04, *p* <.001, partial *η2* = .40), as subjects performed worse as the to-be-recalled string of information lengthened. String Length interacted with gender (*F*(9, 810) = 1.91, *p* <. 05, partial *η2* = .02); as the strings grew longer, the performance of female participants deteriorated. A String Length x Experiment x Gender interaction (*F*(18,810) = 1.9, *p* = .01, partial *η2* = .04) suggests this is because female participants’ performance decrement at long strings was found to a different extent in each experiments. Indeed, the performance delta at T10 (male participants—female participants) was 3.5% for Space Only, 16% for Non-Symbolic Number, and-1.4% for Symbolic Number.

### Spatial Flow as a Strategic Scaffold for Poor Performers

To address the possibility that subjects who are having difficulty with the task selectively utilize structure (either generic, or specific to the MNL) to enhance recall, we sorted the subjects into Low performers (the bottom half of overall percentage correct for subjects in the same experiment) or High performers (the top half). The subjects’ scores were entered in an ANOVA with Spatial Type (LR, RL, IND) as a within-subject factor and gender (female, male), Experiment (Space Only, Non-Symbolic Number, Symbolic Number), and Performance level (High, Low) as between-subjects factors. (We had no strong predictions with respect to the impact of String Length; performance for each spatial type was averaged across this dimension.) The strongest version of this “strategic when struggling” account (considered an alternative to an account based on gender differences in Spatial Type memory) predicts that the gender differences from the previous omnibus ANOVA will disappear in this model, and the variable of Spatial Type will interact with Performance instead. (For the sake of brevity, any noted significant modulations of the data in interactions were established using pairwise comparisons corrected for multiple comparisons.) There was a main effect of Spatial Type (*F*(2,168) = 54.65, *p* <. 001, partial *η2* = .39); LR (93%) and RL (93%) were similarly high relative to IND (87%). The previous simple interaction of Spatial Type with Gender is no longer significant. Spatial Type did interact with Performance Level (*F*(2,168) = 16.26, *p* <.001, partial *η2* = .16); High and Low performers both benefited from LR and RL structure relative to IND (respectively, High Performers: 97%, 97%, 95%; Low Performers: 88%, 88%, 79%), but this benefit was higher for Low performers. There was a Spatial Type x Performance Level x Gender interaction (*F*(2,168) = 3.23, *p* = .04, partial *η2* = .04). A significant quadratic trend in planned contrasts indicates this is related to modulation in RL performance; indeed, low-performing males were significantly better when recalling RL trials relative to low-performing females (92% vs. 85%). Thus, the strategic scaffold hypothesis receives overall support, although it does not entirely account for the gender effects found amongst performance for different spatial types.

## Summary and Concluding Discussion

The experiments detailed within this study utilize several different types of stimuli, alongside multiple manipulations within each experiment. Below we provide a summary and discussion for each conclusion when considering the entire set of experiments.

### Horizontal spatial structure significantly enhanced the ability of subjects to recall spatial locations, especially when the task was more difficult or subjects were poor performers

These results are consistent with other work that examined the role of spatial structure in serial-spatial tasks [26, 39, 40] and the use of strategy choice based on problem difficulty [29, 30]. For example, previous work has found that performance differences for typical Corsi strings in the literature were due to differences in the spatial proximity of each string’s items [39]. Further, specifically manipulating the spatial proximity of subsets of the string (creating “clusters” of information) yields better performance within clusters relative to between clusters [40]. For the structured trials in the current task, the possibility space of where new information should be encoded or recalled was smaller, which likely resulted in a narrower distribution of visual attention and greater attentional resolution [41, 42]. This phenomenon was especially apparent when the task was a challenge; poor performers exhibit a benefit from structure that was four times greater than that of good performers. Further, subjects who had to recall locations under high cognitive load (Experiment 3B) showed the largest structure advantage, another indication that adults here were more likely to use efficient encoding processes when they were most challenged.

### Symbolic number scaffolds spatial memory in a way that other types of stimuli do not

Explicit numerical information tagged to spatial locations resulted in better recall, especially when compared to non-symbolic numerical information, irrespective of MNL congruency. This is potentially due to the use of the cardinal number to keep track of how many items they were asked to recall at test, leading to fewer missed panels or prematurely shortened recall strings. Since the current paradigm allows us to quantify not only relative “hot” and “cool” spatial processing locations on the visual field (located in the upper corners and lateral sides of the screen; Fig. 5), we can observe how this processing shifts as a function of the to-be-remembered stimuli. These findings clearly suggest that adults’ spatial processing in the presence of symbolic number—relative to straightforward spatial locations—increases for nearly the entire visual field, and especially so for those locations that were previously very weak (e.g., the lower right corner of the screen). Thus it appears that this cardinal number process was a useful process to enhance memory. It is noteworthy that subjects did not adopt it independently in the other two conditions, despite the fact that it would have been relatively easy to tag each location with a number and draw the cardinal number benefit. This may be due to the explicit nature of the symbols; students are accustomed to ordering with numerals in their day-to-day life and thus it was a readily available strategy.

### Male participants performed better overall than female participants, especially when the memory load was high

This phenomenon has been found in the working memory literature starting in childhood [43–45]. We did not find that female participants and male participants differed on their dependence of spatial structure, however, suggesting that for this particular paradigm it is overall increased WM span that may have differed between the groups and not a difference in use of directional cues—a difference sometimes found in other types of spatial memory tasks [46, 47].

The relationship between gender, math anxiety, working memory, and mathematics performance is an important topic in the literature. Recent evidence suggests that math anxiety and decrements in visuo-spatial working memory are not only related to one another, but may be mediating factors of the relation between gender and math performance in adulthood and early in life [48, 49]. In the current investigation, gender differences in terms of overall accuracy only emerged in the Non-Symbolic Number and the Symbolic Number with High Cognitive Load experiments. Gender differences were not the subject of this investigation, and we did not specifically explore the impact of the potential candidate mechanisms underlying this performance difference (proposed to range from the biological to the sociocultural.) However, given the importance of understanding the mechanisms—both high- and low-level—that support spatial memory and mathematics, future research should explore this possibility deliberately in more depth.

### A culturally consistent spatial flow of numerical information and MNL congruency impacted performance, but this effect was limited and not documented across conditions

When subjects were tasked with recalling solely spatial information that flowed horizontally, there was a benefit when locations appeared from right-to-left. This benefit was attenuated when symbolic numbers were embedded in the spatial locations, and even reversed (for poor-performing female participants) when non-symbolic numbers were embedded in the spatial locations. In this case, the predicted benefit of left-to-right spatial flow emerged. These differences are in line with work that finds discrepant processing of symbolic and non-symbolic number [50].

One of the most interesting aspects of the gender differences found in this paradigm is that they suggest that female participants are more attuned than male participants to MNL congruency, at least in the context of non-symbolic quantities. For every string length in Experiment 2 female participants showed better memory for left-to-right presented stimuli, and they also were more likely than male participants to exhibit a LVA in the context of MNL congruency. The analysis over all experiments suggests that male and female participants significantly differed with respect to which type of spatial flow was beneficial, with male participants consistently exhibiting a slight benefit for information presented right-to-left and female participants showing a similar benefit for left-to-right. Many researchers (who also found shifts in visual attention as a result of non-symbolic quantities [4]) do not report any analyses involving gender characteristics, so it unclear whether that exerts an effect on these types of studies. This factor is worth considering in future work in the spatial-numerical subfield, especially given the potential role of working memory—and its complex gender differences—on forming spatial-numerical associations.

In confluence with gender effects, we see some evidence that poor performers overall (regardless of gender) are using scaffolds such as the mental number line as a reference when encountering a difficult task. To experimentally address the question of the degree to which this phenomenon is precipitated by poor performance or issues related to gender-based stereotype threat, one would need to control for the subjects’ performance on other SNARC-like tasks or explicitly prime the subject on their gender, neither of which were done in this study. Due to the need to further explore the mechanisms behind sex differences in critical STEM fields, this limitation should be addressed in subsequent research.

One potential reason for the lack of an overall impact of spatial-numerical associations on memory in this task is the occupation of the spatial portion of the subjects’ working memory systems. This accords with work in which sub-types of working memory have been implicated in the presence of different types of spatial-numerical mappings [51–53]. For example, when visuo-spatial working memory and verbal working memory loads are manipulated, the spatial load interfered with magnitude-based SNARC effects [52]. It is possible here that the majority of working memory resources were devoted to spatial location recall, and even though this was happening concurrently to numerical processing it may have taken precedence over linking space and number.

In summary, the experiments from this study reveal that different types of spatial structure, and different types of spatial-numerical associations, influence spatial learning and memory. There were distinct roles in this paradigm for non-symbolic number and symbolic number when encoding and recalling spatial information, with non-symbolic quantities more likely to evoke spatial-numerical associations. Female participants were more likely to show effects of spatial-numerical associations than males. We also find that spatial structure enhances spatial recall regardless of the directional flow of this structure, and that male participants were better able to recall spatial information relative to female participants. We also document the uneven spread of visual processing during a visuo-spatial working memory task, and find that processing concurrent symbolic numbers enhances overall spatial processing. This work supports the view that space and number relate to and scaffold each other in a learning process, and that the nature of this relation is mediated by the individual’s performance level, culture, and gender.

## Supporting Information

S1 Dataset Supplementary file containing all subjects’ data. This spreadsheet gives the raw means for each subject at each location (A is the upper left corner, with lettering moving from left to right and top to bottom), and for each string length in each spatial type.
(XLS)
